# Advances in nanomaterials as novel elicitors of pharmacologically active plant specialized metabolites: current status and future outlooks

**DOI:** 10.1039/c9ra08457f

**Published:** 2019-12-05

**Authors:** Sumaira Anjum, Iram Anjum, Christopher Hano, Sidra Kousar

**Affiliations:** Department of Biotechnology, Kinnaird College for Women Lahore Pakistan sumaira.anjum@kinnaird.edu.pk +92-300-6957038; Laboratoire de Biologie des Ligneux et des Grandes Cultures, INRA USC1328, Université d'Orléans 28000 Chartres France; Department of Chemistry, University of Agriculture Faisalabad Pakistan

## Abstract

During the last few decades major advances have shed light on nanotechnology. Nanomaterials have been widely used in various fields such as medicine, energy, cosmetics, electronics, biotechnology and pharmaceuticals. Owing to their unique physicochemical characteristics and nanoscale structures, nanoparticles (NPs) have the capacity to enter into plant cells and interact with intracellular organelles and various metabolites. The effects of NPs on plant growth, development, physiology and biochemistry have been reported, but their impact on plant specialized metabolism (aka as secondary metabolism) still remains obscure. In reaction to environmental stress and elicitors, a common response in plants results in the production or activation of different types of specialized metabolites (*e.g.*, alkaloids, terpenoids, phenolics and flavonoids). These plant specialized metabolites (SMs) are important for plant adaptation to an adverse environment, but also a huge number of them are biologically active and used in various commercially-valued products (pharmacy, cosmetic, agriculture, food/feed). Due to their wide array of applications, SMs have attracted much attention to explore and develop new strategies to enhance their production in plants. In this context, NPs emerged as a novel class of effective elicitors to enhance the production of various plant SMs. In recent years, many reports have been published regarding the elicitation of SMs by different types of NPs. However, in order to achieve an enhanced and sustainable production of these SMs, in-depth studies are required to figure out the most suitable NP in terms of type, size and/or effective concentration, along with a more complete understanding about their uptake, translocation, internalization and elicitation mechanisms. Herein, we are presenting a comprehensive and critical account of the plant SMs elicitation capacities of the three main classes of nanomaterials (*i.e.*, metallic NPs (MNPs), metal oxide NPs (MONPs) and carbon related nanomaterials). Their different proposed uptake, translocation and internalization pathways as well as elicitation mechanism along with their possible deleterious effect on plant SMs and/or phytotoxic effects are summarized. We also identified and critically discussed the current research gaps existing in this field and requiring future investigation to further improve the use of these nanomaterials for an efficient production of plant SMs.

## Abstract 2

Advancement in nanomaterials as novel elicitors of plant's specialized secondary metabolites.

## Graphical abstract

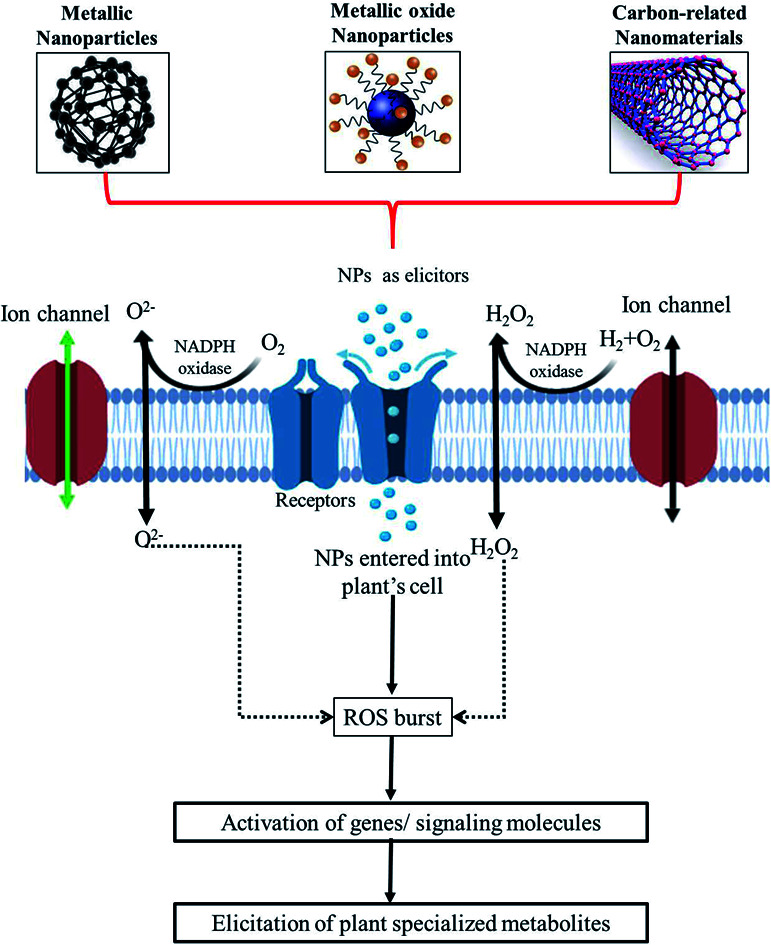

## 1. Introduction

In the last few decades, advances in the field of nanotechnology have signaled major achievements and the use of nanomaterials has extensively progressed. Nanomaterials, including nanoparticles (NPs), dendrimers, nanocoatings, nano-composites, nano-emulsions, nanotubes, fullerenes, nanosheets, and nanoclusters, have delivered what their bulk material had failed to.^1,2^ Today, nanotechnology has infiltrated almost every discipline of science whether in biology, physics, chemistry, material sciences, engineering, biotechnology or medicine. So that, we are currently entering in the Nano Era, a period during which every aspect of life is connected to nanotechnologies.^3^ Nanomaterials are already in use and around us, for examples in textiles, cosmetics, contraptions, appliances, food or environment applications. Extensive research efforts have been made to investigate the potential applications of NPs within human systems, including targeted drug delivery, gene therapy, tissue engineering, cancer therapy, and treatment for infectious and genetic diseases.^4,5^ However, so far, the application of nanotechnology in plant sciences has received comparatively less interest.

**Fig. 1:**
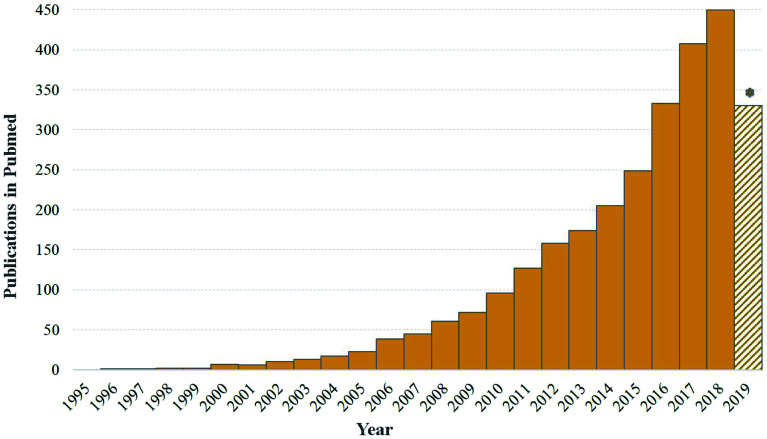
Evolution of publications numbers on nanotechnology and plant occurring in PubMed during the last 25 years. Note that 2019 was still ongoing (accessed on the 12th of August, 2019).

**Fig. 2:**
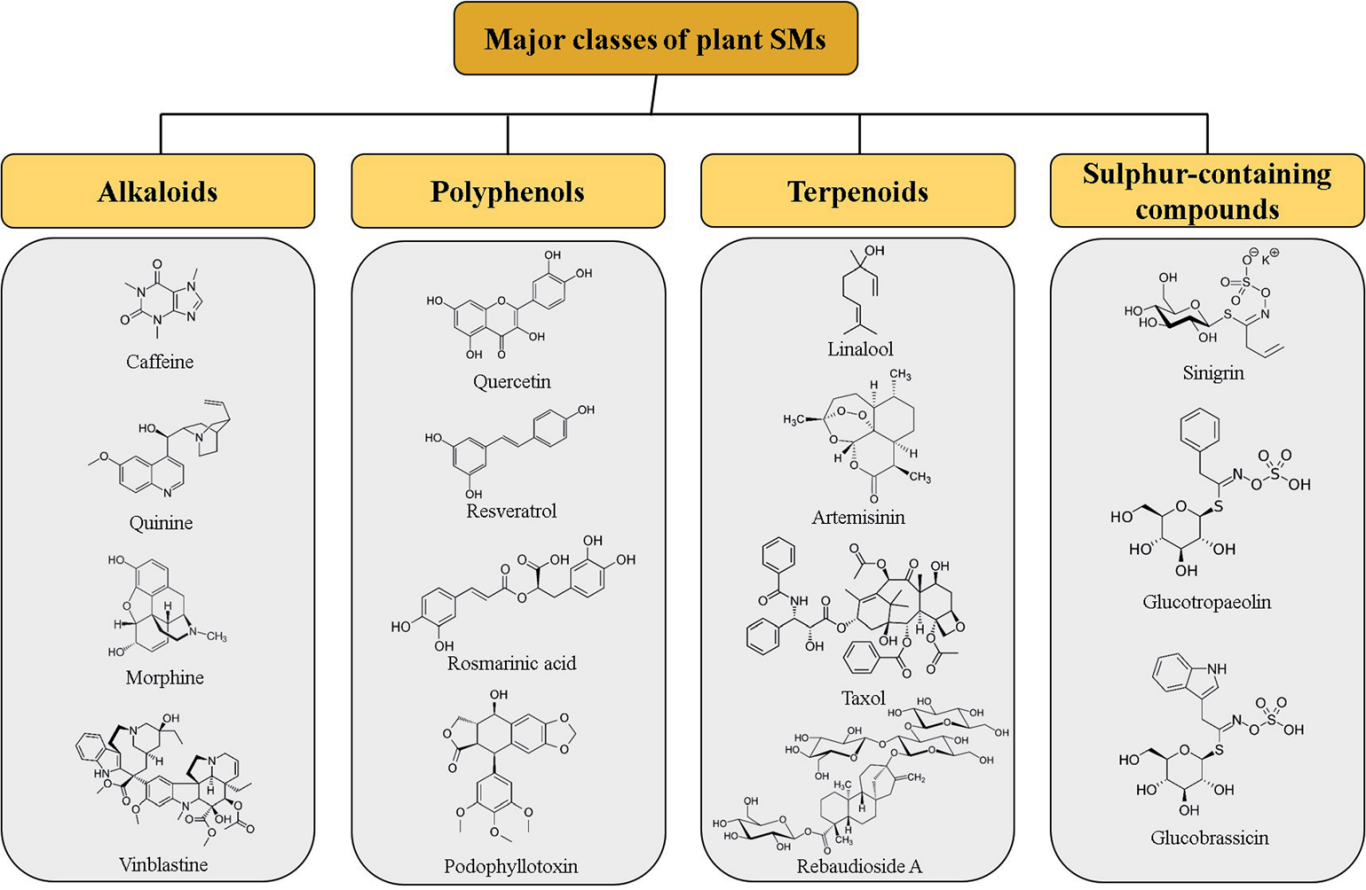
Pharmacologically important specialized metabolites produced in different plant species along with some peculiar examples of each category.

Although, nanotechnology, NPs in particular, has been found to solve many of the agriculture-related tailbacks with significant improvement observed in plant growth of plants, nutrient uptake or plant diseases control as compared to the conventional systems.^6–8^ To date, the majority of the studies have been conducted to evaluate the possible beneficial or toxic effects of NPs on plant growth, development, photosynthesis rate and metabolism, in particular on plant specialized metabolism. Activation and reconfiguration of metabolism during plant acclimation and adaptation to adverse environmental conditions usually involved plant specialized metabolism. Stimulation of specialized metabolism to counteract these environmental stresses, led to the production of different classes of plant SMs,^9,10^ including alkaloids, phenylpropanoids (aka polyphenols), terpenoids and sulphur-containing compounds (including glucosinolates) (Fig. 1), known to act as mediators under both biotic and abiotic stress conditions.^11^ Beside their role in plant defense mechanism and plant adaptation, plant SMs are also used as bioactive compounds in human industries as pharmaceuticals for treatment of various diseases or food and cosmetic additives/ingredients. Some examples of bioactive SMs are given in Fig. 2 including antioxidant (quercetin), antimicrobial (rosmarinic acid), antimalarial (quinine and artemisinin), anti-pain (morphine), cardioprotective and anti-diabetes (caffeine), anti-neurodegenerative (resveratrol), calorie-free sweeteners (rebaudioside A), natural insect repellent (sinigrin, glucotropaeolin, glucobrassicin), *etc.* In particular, currently, the 75% of the pharmaceuticals used for the treatment of cancer originated from plant SMs such as vinblastine, taxol or podophyllotoxin for examples (Fig. 2).^12–14^

The importance of plant SMs in pharmacology and other commercially-valued products, has triggered the development of strategies to enhance their production in plants by exploiting either plant *in vitro* or *in vivo* systems. In the recent years, different strategies such as cell line selection, precursor feeding, cell immobilization, biotransformation, metabolic engineering, synthetic biology and elicitation have been designed to improve the production of valuable plant SMs using plant *in vitro* cultures. Among them, elicitation has emerged as an attractive strategy for enhanced production of pharmaceutically important plant SMs due to its simple implementation and quick responses.^15–17^ To date, different types of biotic and abiotic elicitors have been used for elicitation of plant SMs. Plants respond to these elicitors through the activation of their defense systems, which results in the mobilization of their SMs. In that context, nanotechnology also showed a great potential to elicit plant SMs production, thus providing new tools and new opportunities to develop scalable plant *in vitro* platforms for the production of valuable SMs. Nanotechnology enters the field of plant science about twenty years ago as shown in Fig. 1. The idea of using nanomaterials as elicitors of plant SMs production is even more recent and has emerged in the forefront during the last 5 years with nearly a hundred publications on the subject of which nearly 60% were published in the last two years. Currently, different types of NPs have been used as novel and effective elicitors of SMs in *in vitro* cultures of various plant species.^18–20^ Carbon nanotubes, silver, gold, copper, zinc oxide and titanium dioxide NPs are the most commonly employed types of ‘nano-elicitors’. Significant amounts of data on the manipulation of NPs as novel elicitors of plant SMs has been accumulated in the literature in the recent years (Fig. 1). However, a consolidated interpretation or a critical analysis of these published data is not available to the best of our knowledge. This review includes available literature from the last decade. The search terms or keywords, “Nanomaterials”, “Elicitors”, “Specialized/Secondary metabolism”, “Plant specialized/secondary metabolites”, “Pharmacologically active compounds” and their combinations were used. Here, we holistically review the available data on the elicitation of specialized metabolites by NPs, along with their exposure, uptake, mechanism of elicitation and possible phytotoxic effects on plants. For this purpose, a systematic review of the literature was conducted by searching information published in original articles, technical reports or conference proceedings in scientific databases such as PubMed, Bentham Science, Direct Science Direct Science, Springer, Google Scholar, BMC, MEDLINE, ScopeMed.

## 2. Exposure, uptake and translocation of nanoparticles into plant cells and tissues

**Fig. 3:**
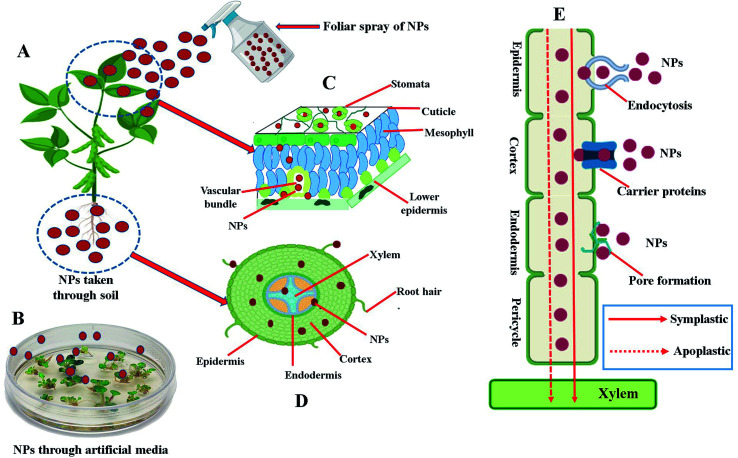
Schematic illustration of the different routes of uptake, entry and translocation of nanoparticles (NPs) into plant cells and organs. (A) Uptake of NPs by the plant either through the leaf by foliar spray or taken through soil. (B) Uptake of NPs by the seeds/tissues/explant growing on artificial prepared nutrient media. (C) Transverse cross section of leaf showing internalization of NPs taken through leaves by foliar spray. (D) Transverse cross section of root showing internalization of NPs taken through roots of plants. (E) Symplastic or apoplastic translocation/movement of the NPs through plant cell.

Owing to their unique characteristics, such as small size, high surface to volume ratio, ability to engineer electron exchange and high surface reactive capabilities, NPs can easily enter and interact with several constituents of plant cells and tissues. Uptake of NPs by plants have been detected and confirmed by using various microscopic/spectroscopic techniques such as transmission electron microscopy, scanning electron microscopy, confocal microscopy, energy dispersive spectroscopy, X-ray fluorescence microscopy, atomic force microscopy, light microscopy, two-photon excitation spectroscopy and Raman spectroscopy.^10,21,22^ Plants can uptake NPs by three main ways: (1) through a foliar spray (Fig. 3A and C), (2) through the soil (Fig. 3D), and (3) through the use of artificially prepared nutrient media (Fig. 3B). To enter into the plant cells and tissue, NPs have to cross a first barrier that is the plant cell wall. The plant cell wall pores, with a diameter usually ranging between 5 to 20 nm in size, can constitute a simple entry way into the plant cells for NPs presenting lesser dimension than the pore diameter.^23^ Some studies also reported the entry of NPs larger than the plant cell wall pore in size either by changing the size of existing plant cell wall pores or by inducting the production of new larger plant cell wall pores.^24–26^ After crossing the plant cell wall, NPs reaches to the cell membrane of plants. From cell membrane, further internalization towards cytosol or other organelles take place either by endocytosis, specific membrane-bound transporter proteins or through induction of new pores by using ion carrier substances.^24,27^ After internalization, NPs can be transported from one cell to another *via* apoplastic or symplastic pathways as shown in Fig. 3E. Following entrance, NPs can interact with various organelles and intracellular components of the plant cells, and potentially disturb both primary and specialized metabolism of plants either by generating a stress through the production of reactive oxygen species (ROS) or by other mechanisms discussed in detail in subsequent headings.

To date, NPs uptake, translocation, accumulation and interaction with plant cells and tissue is relatively a new avenue and a few literature data is available regarding their fate in the plant cell. To comprehensively understand the mechanism of elicitation of specialized metabolite by NPs, it would be very important to characterize in-depth their penetration, translocation and interaction modes within plant cells and tissues.

## 3. Nanomaterials as elicitors of plant specialized metabolites

In this review we have categorized the nanomaterials into three main subclasses: (1) metallic NPs, (2) metal oxide NPs and (3) carbon-related nanomaterials, and discussed their elicitation potential of plant SM.

### 3.1. Metallic nanoparticles

Metallic nanoparticles (MNPs) have been largely employed in different plant species, and their impact on mass propagations, genetic manipulation, elimination of microbial content and production of SM have been reported. Owning to their unique properties, different MNPs (Ag, Cu, Au, Co, Zn) have also been used as elicitors of plant SM in various species (Table 1). The impact of MNPs on SM production was reported to depend on the characteristics of the MNPs (*i.e.*, the concentration used, the exposure time and their size and synthetic origin), but also on the plant culture type.^28–30^ In this review, we have summarized these different parameters that can affect the impact of MNPs on SM production in a number of *in vitro* cultures of crops and/or medicinal plant species.

**Table 1:** Summary of the effects of various types of metallic nanoparticles (MNPs) used as elicitors of specialized metabolites in different plant species

NPs	Size of NPs (nm)	Effective conc. of NPs	Plant species	Culture type	Growth media/conditions	Effect on specialized metabolites	Phytotoxicity	References
Ag	—	8–10 mg L^−1^	*Glycyrrhiza glabra*	Seed culture	MS media	Increase in total quercetin (39%) and glycyrrhizin (23.49 μg g^−1^ DW) contents	—	39
Ag	—	5 ppm	*Corylus avellana*	Cell suspension	—	Increase in taxanes production (taxol, 378% and baccatin III, 126%)	Decrease in the growth of cells	41
Ag	—	5 mg L^−1^	*Momordica charantia*	Cell suspension	MS media with TDZ and 2,4-D	Increase in production of flavanols hydroxybenzoic and hydroxycinnamic	—	28
Ag	—	45 mg L^−1^	*Stevia rebaudiana Bertoni*	Callus	MS media with NAA and BA	Enhanced production of stevia glycosides (stevioside; 67%)	—	30
Ag	40	90 μg L^−1^	*Caralluma tuberculata*	Callus	MS media with BA and 2, 4 D	Increase in total phenolic (3.8 mg g^−1^ DW) and flavonoid (1.8 mg g^−1^ DW) contents		51
Ag	—	—	*Brassica rapa*	Hairy root	MS media	Enhanced production of glucosinolates (approx. 2.9%) (gluconasturtiin, glucobrassicin, 4-methoxyglucobrassicin, neoglucobrassicin, 4-hydroxyglucobrassicin, glucoallysin, glucobrassicanapin, sinigrin, progoitrin, and gluconapin) and phenolic compounds (flavonols, hydroxybenzoic and hydroxycinnamic acids)	—	29
Ag	5–35	40 mg L^−1^	*Pelargonium graveolens*	Seedlings	Potting mix	Almost 14% increase in production of major essential oil constituents (citronellol, geraniol, cirtonellyl formate, isomenthone, linalool and *E*-caryophyllene)	—	50
Ag	30–32	60 ppm	*Thymus kotschyanus*	Plantlets	Potting mix	Enhanced production of essential oils and α-terpinyl acetate content	Reduction in thymol content	43
Ag	—	0.3 mg L^−1^	*Calendula officinalis*	Callus	MS media	Production of essential oils (89.23 mg mL^−1^ DW) was enhanced	—	44
Ag	8–47	50 μM	*Arabidopsis thaliana*	Seedlings	MS media	Increased in production of anthocyanins (56%)	—	47
Ag	15–35	50 mg L^−1^	*Vanilla planifolia*	Shoot culture	MS media	Increased in production of total phenolic contents (78.23 mg g^−1^ QE) and antioxidant enzymes	—	48
Ag	—	500 mg L^−1^	*Ricinus communis* L.	Seed germination	Filter paper	Increased total phenolic content (34%) and antioxidant enzymes concentrations	Inhibition of seed germination	33
Ag	—	900 mg L^−1^	*Artemisia annua*	Hairy root	MS media	3.9 fold (3.31 mg g^−1^ DW) increase in artemisinin production	—	37
Ag	50–60	20 mg L^−1^	*Datura metel*	Hairy root	MS media	Enhancement in the production of atropine (19%)	—	36
Ag	—	0.625 mg L^−1^	*Aloe vera*	Cell suspension	MS media	Increase in production of aloin (127%) contents	—	35
Ag	—	3.0 mg L^−1^	*Capsicum frutescens*	Cell suspension	MS media	Content of capsaicin was increased 2-fold	—	19
Ag	—	5.0 mg L^−1^	*Corylus avellana*	Cell suspension	MS media	Enhanced production of taxol (34%)	Decreased cell viability	42
Ag	—	30 μg L^−1^	*Linum usitatissimum*	Cell suspension	MS media	Enhanced production of lignans (67.23 mg g^−1^ DW) and neolignans (45.9 mg g^−1^ DW)	—	34
Ag	—	2.0 mg L^−1^	*Cucumis anguria*	Hairy root	MS media	4-Fold increase in phenolic compounds (flavonols, hydroxycinnamic and hydroxybenzoic acids), was observed	—	31
Ag	—	40 mg L^−1^	*Oryza sativa*	Seedling	MS media	Carotenoid contents (67%) were increased	—	32
Ag	—	1.0 μg mL^−1^	*Trigonella foenum-graecum*	Seedlings	Agar	Diosgenin production (214.06 ± 17.07 μg mL^−1^) was increased	—	
Ag		25 μg mL^−1^	*Arabidopsis thaliana*	Plantlets	MS media	Anthocyanin and flavonoid production was accelerated	—	49
Ag	40	0.4 mM	*Calendula officinalis*	Plant	Hoagland solution	Saponin contents (177%) were increased	Decrease in total phenolic, flavonoid and anthocyanin contents	40
Ag	—	50 ppm	*Amaranthus caudatus*	Seed germination	Garden soil	Increase in total phenolic (68.19%) and flavonoid (35.21%) contents was observed	—	45
Ag	15–100	10 ppm	*Bacopa monnieri*	Seed germination	Hydroponic culture	Increase in total phenolic (42%) and antioxidant activity (39%) was observed	—	53
Cu	40	20 mg L^−1^	*Cucumis sativus*	Hydroponic culture	Hoagland solution	Increased in total phenolic content (2.35 mg g^−1^ DW)	—	57
Cu	—	5 μM	*Verbena bipinnatifida*	Shoot culture	MS media	Two fold increase in total phenolic content	—	54
Cu	50	50 mg L^−1^	*Solanum lycopersicum*	Plantlets	Green house conditions	64.12% lycopene, 5.43% total phenolic and 26.21% flavonoid contents were increased as compared to control	—	55
Cu	—	0.5 mg L^−1^	*Mentha longifolia*	Shoot culture	MS media	Essential oil contents (91.02%) were increased in response to both type of NP treatments (Cu and Co)	—	56
Co		0.8 mg L^−1^						
Co	10	5.0 mg L^−1^	*Artemisia annua*	Cell suspension	MS media	2.2 Fold increase in artemisinin production	—	38
Cu–Au bimetallic	—	—	*Stevia rebaudiana Bertoni*	Adventitious root culture	MS media with NAA	Total phenolic (54%) and flavonoid (20%) contents were increased	—	59
Ag + Au	Au (24) Ag (27)	10–50 mg dm^−3^	*Lavandula angustifolia*	Cell suspension	MS media	2.3-Fold increase in essential oils production	—	62
Ag + Au	—	1 : 3 ratio	*Prunella vulgaris* L.	Callus	MS media with NAA	Increase in total phenolic (23%) and flavonoid (4%) contents	—	58
Zn + Ag	30	19 : 1 ratio	*Withania somnifera*	Seed germination	Potting soil	Withanolide contents (87.23 mg g^−1^ DW) were enhanced	—	60
Ag + Au	—	1 : 3 ratio	*Prunella vulgaris* L.	Cell suspension culture	MS media with NAA	1.8-Fold increase in total phenolic and flavonoid contents	—	61

#### 3.1.1. Elicitation potential of silver nanoparticles (AgNPs)

Silver nanoparticles (AgNPs) are certainly the most exploited MNPs as elicitors of plant SM in *in vitro* cultures of various plant species.^31–33^

An effective elicitation of plant SMs of AgNPs have been described for many plant species in diverse production systems. For instance in cell suspension systems, AgNPs (30 μg L^−1^) have been described as an effective elicitor stimulating the production of both lignans (67.23 mg g^−1^ DW) (secoisolariciresinol diglucoside and lariciresinol diglucoside) and neolignans (45.9 mg g^−1^ DW) (dehydrodiconiferyl alcohol guaiacylglycerol-β-coniferyl alcohol ether glucoside) in cell suspension of *Linum usitatissimum*.^34^ Cell suspension culture of *Capsicum frutescens* treated with AgNPs (3.0 mg L^−1^) accumulated 2-times more capsaicin.^19^ Largely used for its medicinal and cosmetic applications, aloin (127%) production was significantly increased in *Aloe vera* cell suspension treated with AgNPs (at 0.625 mg L^−1^).^35^ Hairy root systems is one of the most operative *in vitro* platform for production of valuable plant SMs and elicitation experiments by using different MNPs have been reported in hairy root cultures of many plant species.^31,36,37^ In another study, researchers reported a 3.9% fold increase in production of artemisinin in hairy root culture of *Artemisia annua* after treatment with AgNPs (900 mg L^−1^).^37^ Note that a 2.2 fold increase was also observed in root culture of *A. annua* when treated with cobalt nanoparticles.^38^ Increased production of atropine was observed in hairy root culture of *Datura metel* after addition of AgNPs (200 mg L^−1^ of 50–60 nm sized) as an elicitor in the culture medium.^36^ Similarly, enhanced production of flavonoids and phenolic compounds were reported in hairy root culture of *Cucumis anguria* elicited with AgNPs (2.0 mg L^−1^).^31^*In vitro* grown plants were also considered. Quercetin and glycyrrhizin production was increased in seedlings of *Glycyrrhiza glabra* following the addition of AgNPs (8–10 mg L^−1^) to the growth media. Note that in the present case a more complex response was observed with quercetin found in higher level in the aerial parts of the seedlings, while the glycyrrhizin content was found higher in roots.^39^ Similarly, exposure of AgNPs showed both inhibitory and stimulatory effect on SMs production of *Calendula officinalis* growing under *in vitro* condition with a strong decrease in carotenoid content, whereas a 2-fold increase in saponin production was observed.^40^

A particular attention have also to be paid to the AgNPs concentration used. Effect of AgNPs on SM productions is not simple and their impact on plant growth parameters have to be carefully taken into account since growth perturbations or even toxic effects on plants have been reported. A stimulation of taxanes production elicitation in cell suspension culture of hazel cells (*Corylus avellana* L.) was reported recently.^41^ Hazel cells treated with different concentrations of AgNPs (2.5, 5 and 10 ppm) during their exponential growth phase and harvested one week after treatment for analysis. A dose dependent effect of AgNPs was observed with a strong increased production yields of anticancer taxanes in response to 5 ppm concentration of AgNPs: increased by 378% for taxol and by 163% for baccatin III. However, treated cells presented a significant growth reduction. Note that similar positive effect on taxol production in cell suspension culture of the same plant, *Corylus avellana*, was also described following addition of 5.0 mg L^−1^ of AgNPs.^42^ The dose dependent effects of AgNPs elicitation was also reported on the production of terpenes in plantlets of *Thymus kotschyanus* (*i.e.* increased production of α-terpinyl acetate)^43^ and callus culture of *Calendula officinalis* (*i.e.* increased production of essential oils).^44^ Chung *et al.*,^31^ investigating the effect of AgNPs on seed germination rate and phenolics production of *Ricinus communis* reported an ideal concentration of 500 mg L^−1^ to increase both parameters, whereas a higher concentrations have a negative impact on these parameters.^31^ On the contrary, stimulation of antioxidant phenolic compounds production without severe toxic effects was observed in hydroponically grown *Bacopa monnieri* treated with AgNPs.^45^ Gupta *et al.*,^32^ reported the stimulatory effect of AgNPs (10–40 mg L^−1^) on both root and shoot growth along with enhanced production of carotenoid (67%) in seedlings of *Oryza sativa*. AgNPs (1 μg mL^−1^) elicited *in vitro* culture of *Trigonella foenum graecum* showed enhanced production of diosgenin (214.06 ± 17.07 μg mL^−1^) associated with a significant increase in plant growth.^46^

The size and shape of these MNPs have been shown to affect various growth parameters of plants along with the elicitation of SMs. For instance, spherical (8 nm), decahedral (32 nm) and triangular (47 nm)-sized AgNPs were used at the same concentration of 50 μM showed contrasting results.^47^ Contrary to decahedral and triangular AgNPs treated-plants, *A. thaliana* seedlings treated with spherical AgNPs showed enhanced accumulation of anthocyanin without any negative effect on root growth. Similarly, the effect on shoot culture of *Vanilla planifolia* exposed to different sizes (15–35 nm) of AgNPs, showed that smaller size AgNPs (18 nm) significantly enhanced production of phenolic compounds (78.23 mg g^−1^ QE) as compared to larger sized nanoparticles.^48^*A. thaliana* plantlets treated with spherical shaped AgNPs (25 μg mL^−1^) ranging in size between 20 to 30 nm showed enhanced production of anthocyanin, total flavonoid and phenolic contents.^49^

From a mechanistic point of view, most of the studies reported that the generation of reactive oxygen species (ROS) following exposure to MNPs as the central basis for their plant SMs elicitation capacity.^29,42,50^ ROS are known to behave as signaling molecule in the management of plant defense response under both biotic and abiotic stress that could result in the production stimulation of stress-responsive SMs.^29^ reported that the AgNPs exposure to hairy root culture of *Brassica rapa* resulted in an active generation of ROS (hydrogen peroxide) which in turn activate the plant defense mechanism with an enhanced production of glucosinolates (2.9%) (glucoallysin, neoglucobrassicin, glucobrassicanapin, gluconapin, 4-methoxyglucobrassicin, sinigrin, 4-hydroxyglucobrassicin, glucobrassicin, progoitrin and gluconasturtiin), phenolic and flavonoid compounds. Similarly, the generation of ROS after exposure to on exposure of AgNPs (5–35 nm, 40 mg L^−1^) resulted an increased content of essential oil components such as geraniol, citronellyl formate, and *E*-caryophyllene in *Pelargonium graveolens* seedlings.^50^

A possible interaction with plant growth regulators have also been proposed. Plant growth regulators are not only essential for plant growth and development but also effect the production of SMs. Plant growth regulators have been employed along with different MNPs to check their combinative effect on elicitation of SMs in various plant species.^28,51,52^ In combination with plant growth regulators (TDZ and 2,4-D), AgNP elicitation of bitter gourd (*Momordica charantia*) cell culture resulted in an enhanced production of phenolic, hydroxycinnamic and hydroxybenzoic acids, and flavonoids.^53^ Enhanced production of stevia glycosides was observed in callus culture of *Stevia rebaudiana Bertoni* simultaneously elicited with AgNPs and salicylic acid.^52^ Similar results were reported by Ali *et al.*,^51^ upon treatment of callus culture *Caralluma tuberculata* with AgNPs along with BA and 2,4 D resulting in an increase productions of total phenolics (3.8 mg g^−1^ DW) and flavonoids (1.8 mg g^−1^ DW). Note that in this later case, an increase in callus proliferation and biomass was also reported.

#### 3.1.2. Elicitation potential of copper nanoparticles (CuNPs)

Copper nanoparticles (CuNPs) are used in agricultural science and plant nanotechnology in order to improve growth, yields as well as SMs production. Genady *et al.*,^54^ showed that CuNPs (5 μM) are efficient to enhance the production of total phenolic (2-fold) in shoot culture of *Verbena bipinnatifida*. Another report also suggested the positive effect of CuNPs (50 mg L^−1^) on the SM production in shoot culture of *Solanum lycopersicum*, with increased accumulations of lycopene (64.21%), total phenolic (5.43%) and flavonoid (26.21%).^55^ Similarly, a significant increase in essential oil contents following CuNPs (0.5 mg L^−1^) treatment in shoot culture of *Mentha longifolia* was reported by.^56^ Note that in the same study, the Authors have also reported on a similar effect for cobalt (0.8 mg L^−1^) NPs treatment. However, both constructive and destructive effects on plant growth and SMs production of CuNPs treatments have been reported for cucumber plant grown under hydroponic conditions.^57^ The results suggested the activation of defense mechanism following CuNPs treatment and highest metabolic perturbations associated with enhanced production of total phenolic contents were observed in roots in contact with CuNPs.

#### 3.1.3. Elicitation potential of combination of different MNPs/bimetallic nanoparticles

Bimetallic nanoparticles and different combinations of MNPs have also been reported as effective elicitors of SMs production in various plant species.^58–60^ The effect of Ag and Au NPs in combinations (1 : 2, 1 : 3, 2 : 1, and 3 : 1) or separately on callus cultures of *Prunella vulgaris* L., showed that the application of a 1 : 3 ratio of Ag : Au NPs significantly increased the total flavonoids (4%) and phenolics (23%) productions.^58^ More recently, the same authors showed that this 1 : 3 ratio of Ag : Au NPs was also the most efficient in enhanced production of total phenolic and flavonoid contents in a cell culture system of the same species (*i.e. P. vulgaris* L.).^61^ Cu–Au bimetallic NPs (in a 3 : 1 ratio) were reported as effective elicitors for enhanced production of total phenolic (54%) and flavonoid (20%) contents in adventitious root culture of *Stevia rebaudiana*^59,60^ studying the effect of different ratios (19 : 1, 3 : 1, 9 : 1 and 1 : 1) of zinc (Zn) and AgNPs on SMs production of *Withania somnifera* grown under different conditions, proved that the 19 : 1 ratio was the most effective in enhancing the withanolide content, through the activation of ROS production. A more complex effect was reported for different combinations of Ag and Au NPs used as elicitors of essential oil production in cell suspension culture of *Lavandula angustifolia*, with a decreased accumulation of low molecular weight components (such as trans-pinocarveol and 1,8-cineole), whereas a concomitant increase in high molecular weight compounds (such as cadalene) was observed.^62^

### 3.2. Metal oxide nanoparticles

The literature provides assorted, and often contradictory results on the plant responses exposed to different metal oxide nanoparticles MONPs. Numerous studies have been carried on the elicitation behaviour of different MONPs in *in vitro* cultures of various plant species.^63–66^ The most widely used MONPs as elicitors of SMs are copper oxide (CuO), zinc oxide (ZnO), titanium oxide (TiO_2_), cesium oxide (CeO_2_), cadmium oxide (CdO) and aluminum oxide (Al_2_O_3_) NPs. Impact of different MONPs on SMs of various plant species is summarized in Table 2. We have also discussed the nonmetallic oxide NPs of silica oxide (SiO_2_) in this subheading.

**Table 2:** Summary of the effects of different types of metal oxide nanoparticles (MONPs) and non-metal oxide nanoparticles (only SiO_2_NPs) used as elicitors of specialized metabolites in different plant species

NPs	Size of NPs (nm)	Effective conc. of NPs	Plant species	Culture type	Growth media/conditions	Effects on specialized metabolites	Phytotoxicity	References
CuO	—	1 ppm	*Withania somnifera*	Shoot and root	MS media	Total phenolic (27.31 mg g^−1^ DW), flavonoid (91.11 mg g^−1^ DW), and tannin (32.02 mg g^−1^ DW), contents were increased	—	70
CuO	25–55	3 mg L^−1^	*Gymnema sylvestre*	Cell suspension	MS media	2-Fold increase in production of phenolic and flavonoids whereas 2.3 fold increase in gymnemic acid	—	64
CuO	—	—	*Brassica rapa*	Hairy root culture	—	14% increase in production of glucosinolates (gluconasturtiin, glucobrassicin, 4-methoxyglucobrassicin, neoglucobrassicin, 4-hydroxyglucobrassicin, glucoallysin, glucobrassicanapin, sinigrin, progoitrin, and gluconapin) and phenolic compounds (flavonols, hydroxybenzoic and hydroxycinnamic acids)	—	71
CuO	—	1 ppm	*Cichorium intybus*	Shoot and root	MS media	Total phenolic (28.171 mg g^−1^ DW), flavonoid (9.450 mg g^−1^ DW) contents were increased	—	72
CuO	47	5 mg L^−1^	*Trigonella foenum-graecum*	Seed germination	MS media	Total phenolic (3.2 μg QE per mg DW) and flavonoid (3.7 μg QE per mg DW) contents were enhanced	Shoot and root growth was inhibited at higher concentration (400 mg L^−1^)	69
CuO	25–55	—	*Brassica rapa*	Seedlings	—	Increased level of reactive oxygen species (ROS), hydrogen peroxide, malondialdehyde (MDA), glucosinolate, proline, anthocyanin (1.3 fold) and phenolic (1.1-fold) compounds	Concentration of chlorophyll, sugar and carotenoid decreased	63
ZnO	—	300, 500 ppm	*Solanum tuberosum*	Plants	Potting soil	Total phenolic (99.1 mg g^−1^ FW) and anthocyanin (3.28 mg g^−1^ FW) contents were increased	—	77
ZnO	—	25–100 mg L^−1^	*Lilium ledebourii*	Shoot	MS media	Enhanced production of total flavonoid (25 mg L^−1^) phenolics (75 mg L^−1^) and anthocyanin (100 mg L^−1^)	—	73
ZnO		75 mg L^−1^	*Echinacea purpurea*	Callus	MS media	Increase in total flavonoid (23.25 mg g^−1^ DW) content	—	76
ZnO	34	1.0 mg L^−1^	*Stevia rebaudiana*	Shoot	MS media	Increased production of total phenolic, flavonoids and steviol glycosides (88.21 mg g^−1^ DW)	—	78
ZnO	—	100 mg L^−1^	*Hyoscyamus reticulatus*	Hairy root	MS media	Production of tropane alkaloids (1.2 fold) and total phenolic contents (3.2 fold) were increased	—	18
ZnO	—	1 μM	*Glycyrrhiza glabra*	Seedling growth	Hoagland's solution	Both ZnO and CuO resulted in increased content of glycyrrhizin, phenolic, flavonoids, anthocyanins and tannins	—	75
CuO		1 μM						
ZnO	—	100 mg L^−1^	*Stevia rebaudiana*	Callus	MS media	Both ZnO and CuO treatments resulted in increase of total phenolic (5.06 lg mg^−1^ of DW), and flavonoid (2.23 lg mg^−1^ of DW) contents	—	79
CuO		10 mg L^−1^						
ZnO	18–20	100–1000 mg L^−1^	*Solanum melongena*	Seed germination	Filter paper	Total phenolic, flavonoid and anthocyanin contents were increased in a dose-dependent manner in response to all three types of NPs	Seedling growth was suppressed	80
NiO	10–20							
CuO	25–55							
Fe_3_O_4_	—	450, 900 mg L^−1^	*Hyoscyamus reticulatus*	Hairy root	MS media	5-Fold increase in production of hyoscyamine and scopolamine	—	20
Fe_3_O_4_	20	30 ppb	*Lepidum sativum*	Plants	Field conditions	Increase in total essential oil (133%), phenolic (13027 mg GAE per g), and flavonoid (453 mg QE per g) contents	—	81
Fe_3_O_4_	—	100 ppb	*Hypericum perforatum*	Cell suspension	MS media	Both ZnO and Fe_2_O_3_ nanoparticles resulted in enhanced production of hypericin (3-fold) and hyperforin (13-fold)	—	83
ZnO								
Fe_3_O_4_	—	50 mg L^−1^	*Cichorium intybus*	Hairy root	MS media	Total phenolic (4.65 mg g^−1^ DW) and flavonoid (77.34 μg g^−1^ DW) contents were increased in response to Fe_3_O_4_NPs while ZnONPs have no significant effect on specialized metabolites production	—	82
ZnO		50 mg L^−1^						
TiO_2_	10–15	100, 200 mg L^−1^	*Salvia officinalis*	Seedlings growth	Potting mix	Monoterpenes (camphene, *p*-cymene, 1,8-cineol, *cis*-thujene, camphor) and total phenolic (35.2 mg GAE per g DW) and flavonoid (21.9 mg CE per g DW) contents were enhanced in response to 200 mg L^−1^ and 100 mg L^−1^ concentration respectively	—	90
TiO_2_	10–15	20, 80 mg L^−1^	*Hyoscyamus niger*	Plantlets	Potting mix	Enhanced production of tropane alkaloids (hyoscyamine; 0.286 g kg^−1^ and in response to 20 mg L^−1^ and scopolamine; 0.126 g kg^−1^) in response to 80 mg L^−1^ conc.	—	91
TiO_2_	—	6.0 mg L^−1^	*Cicer arietinum*	Embryonic callus	MS media	Increased production of phenolic compounds such as gallic acid, chlorogenic acid, *o*-coumaric acid, tannic acid and cinnamic acid	—	89
TiO_2_	—	120 mg L^−1^	*Aleo vera*	Cell suspension	MS media	Aloin contents (118%) were increased	—	35
TiO_2_	25	50, 100 ppm	*Dracocephalum moldavica*	Field cultivation	—	Luteolin 7-*O*-glucoside (2.1 mg g^−1^ FW), rosmarinic acid (0.32 mg g^−1^ FW), *p*-cumaric acid (8.9 mg g^−1^ FW), ellagitannin (4.3 mg g^−1^ FW), gentisic (10.21 mg g^−1^ FW), chlorogenic acid (32.12 mg g^−1^ FW), and caffeic acid (8.23 mg g^−1^ FW) concentration were increased	Gentisic acid contents were reduced	92
CeO_2_	8	250 mg kg^−1^	*Raphanus sativus*	Seed germination	Potting soil	32% increase in antioxidant compounds but no effect on total phenolic and flavonoid contents		95
CeO_2_	33.05	0.1 mM	*Solanum lycopersicum*	Hydroponic culture	Hoagland solution	37% increase in total chlorophyll and 26% in carotenoid content was observed	—	96
CeO_2_	—	1000 mg L^−1^	*Arabidopsis thaliana*	Plantlets	Potting mix	Antioxidant enzymes (superoxide dismutase, catalase, ascorbate peroxidase, and peroxidase) concentrations were elevated along with phenolic (27%) compounds	—	97
In_2_O_3_								
Al_2_O_3_	50	40 μg mL^−1^	*Nicotiana tabacum*	Cell suspension	MS media	Increase in total phenolic contents (62%)	Decreased cell density and viability	98
CdO	7–60	2.03 × 10^5^ particles cm^−3^	*Hordeum vulgare*	Plantlets	Soil, water	Ferulic acid (30%) and isovitexin (183%) were increased	—	99
Mn_2_O_3_	—	25 mg L^−1^	*Atropa belladonna*	Shoot-tip	MS media	Increase in production of alkaloids (23%), total phenolic (12%) and flavonoid (32%) contents	—	100
SiO_2_	100		*Dracocephalum kotschyi*	Hairy root	MS media	Almost 2-fold increase in rosmarinic acid, xanthomicrol, isokaempferide and cirsimaritin increased	—	101
SiO_2_	—	100 mg L^−1^	*Nigella sativa*	Plants at flowering stage	Foliar spray	Elicitation of thymoquinone (2.9 mg g^−1^ DW) production by up-regulating the expression of geranyl diphosphate synthase gene	—	102
TiO_2_		100 mg L^−1^						

#### 3.2.1. Elicitation potential of copper oxide nanoparticles (CuONPs)

Copper (Cu) is an essential element for plant nutrition, and plays a pivotal role in both primary and specialized metabolism of plants. Numerous studies have been published on the impacts of both Cu deficiency and excess on these aspects,^67,68^ but only limited information about the effects of CuONPs are available. CuONPs have been proposed as elicitor of valuable bioactive compounds in a bioreactor systems,^69^ but from literature review specific optimizations to the selected plant species and targeted SM(s) production appear as a critical prerequisite. The efficiency of CuONPs (1 ppm) as elicitors of SMs [total phenolic (27.31 mg g^−1^ DW), flavonoid (91.11 mg g^−1^ DW), and tannin (32.02 mg g^−1^ DW)] production was also assessed by using shoot and tip culture of *Withania somnifera*.^70^ In the same way, different concentrations of CuONPs (1, 3 and 5 mg L^−1^) were effectively used to enhanced the production of SMs (phenolics, flavonoids and gymnemic acid) in cell culture of *Gymnema sylvestre*.^64^ Chung *et al.*,^63^ reported on SMs accumulation but also on the physiological biochemical and transcriptional changes in seedlings of *Brassica rapa* subjected to CuONPs (25–55 nm) treatment. CuONPs treatment resulted in a significant increase in anthocyanin (1.1-fold) and phenolic (1.3-fold) accumulations, together with a stimulation of ROS production. However, a negative effect on primary metabolism was observed as evidenced by chlorophyll content and sugar production decreases. This result was confirmed in hairy root culture of the same plant (*Brassica rapa*) treated with CuONPs showing significantly increased productions of phenolic compounds (hydroxycinnamic acids, flavonols and hydroxybenzoic acid) as described by,^63^ but also of glucosinolates (glucobrassicin, gluconasturtiin, 4-meth-oxyglucobrassicin, 4-hydroxyglucobrassicin, neoglucobrassicin, glucobrassicanapin, glucoallysin sinigrin, gluconapin and progoitrin) accumulated in high amount in this system.^71^ The effect of CuONPs (1 ppm) for the elicitation of polyphenolic content in shoot culture of chicory (*Cichorium intybus* L.) investigated by^72^ showed a clear difference in the timing production of the SMs: a significant increase in total phenolic and flavonoid contents was noted after 20 days of nanoparticle treatment, whereas the tannin contents were higher (6-fold) in 10 days-treated shoots and roots of chicory.^69^ studied the effect of CuONPs (5 mg L^−1^) on seed germination as well as callus induction *Trigonella foenum-graecum*. Low concentrations of CuONPs resulted in root and shoot elongation but the higher concentrations (4000 mg L^−1^) suppressed the root development. If there was no obvious effect of CuONPs on seed germination rate, however the used of capped NPs reduced the toxicity. The production of flavonoids and phenolics increased in both roots and shoots.

#### 3.2.2. Elicitation potential of zinc oxide nanoparticles (ZnONPs)

Zinc oxide nanoparticles (ZnONPs) have vast array of applications due to their unique optical, catalytic, band gap, and high surface area to volume ratio properties. Their impact on plant SMs was studied in various plant species.^18,73–75^ However, unpredictable results were commonly found in different studies about the interaction of ZnONPs with primary and specialized metabolisms of plants.

The effectiveness of ZnONPs to stimulate plant SMs production was reported in several plant species and culture systems. The total flavonoid contents were increased in callus culture of *Echinacea purpurea* in response to a 75 mg L^−1^ treatment with ZnONPs.^76^ Similarly, ZnONPs were found to increase the total phenolic (99.1 mg g^−1^ FW) and anthocyanin (3.28 mg g^−1^ FW) contents in potato plants when applied at 300 and 500 ppm concentration in media, respectively.^77^ But the response to ZnONPs treatment appears specific to the class of plant SMs considered. Treatment of hairy root culture of *Hyoscyamus reticulatus* with ZnONPs at different concentrations (0, 50, 100 and 200 mg L^−1^) revealed a positive effect on the production of both phenolic compounds (3.2-fold) and tropane alkaloids (1.2-fold increase in scopolamine and hyoscyamine). Interestingly, a positive action on the growth rate of the treated hairy root culture was also observed, which is of particular interest considering the action of these phytochemicals used to treat Alzheimer's disease by acting as inhibitors of parasympathetic nervous system.^18^ On the contrary,^78^ using *in vitro* grown shoots of *Stevia rebaudiana* reported on both favorable and adverse actions of ZnONPs on diverse classes of SMs. Indeed, their results showed a significant increase in steviol glycosides production (88.21 mg g^−1^ DW) in micro-propagated shoots treated by ZnONPs (1 mg L^−1^). However, the total phenolic and flavonoid contents were decreased in the same tissue under the same conditions. In another study, the same authors compared ZnONPs and CuONPs as elicitors of steviol glycosides in callus culture of *S. rebaudiana*.^79^ They reported a higher production of steviol glycosides, phenolics and flavonoids in response to ZnONPs than to CuONPs, certainly as a consequence of the toxic effects observed with CuONPs. But this toxic effect is certainly dependent on the plant species since^75^ reported a significant increase in the accumulation of glycyrrhizin, phenolics, anthocyanins, tannins and flavonoids in 21 days old seedling of *G. glabra* using a higher concentration of CuONPs (10 μM) than ZnONPs (1 μm) for the treatment, thus suggesting less toxic effect of CuONPs on this plant species.

Once again, as a first step for the use of these NPs, the applied concentration on the plant culture appears as an important parameter that have to be precisely optimized. For instance, total phenolics, anthocyanins and total flavonoids contents were affected in dose-dependent manner in response to three different types of MONPs (CuONPs, ZnONPs and NiONPs). In this study, different concentrations (100, 250, 500 and 1000 mg L^−1^) of MONPs were used to elicit SMs production in seedlings of *Solanum melongena*. Best results on SMs production were obtained with CuONPs at a concentration level of 500 mg L^−1^, but some negative effects where observed on plant growth as well as viability, mainly due to the generation of high levels of ROS in response to NPs. If the toxic effects are subsided, these MONPs, CuONPs in particular, could be a source of useful specialized metabolites.^80^ One possibility to cope with this toxic effect, could be the association with humic acid as suggested by.^73^ These Authors reported on the impact of ZnONPs along with humic acid on *Lilium ledebourii* plantlets. Once again a ZnONPs dose dependent response for the production of SMs was observed, with maximum production of phenolic acids (75 mg L^−1^) and anthocyanins (100 mg L^−1^) obtained at 75 mg L^−1^ of ZnONPs, whereas highest flavonoid content (25 mg L^−1^) were obtained with 25 mg L^−1^ of ZnONPs. Interestingly, explants treated with humic acid gave attractive results on plant growth parameters such as highest root length, leaf length, and chlorophyll contents. It was suggested that both the humic acid and ZnONPs might be used as good elicitors and can probably stimulate the synthesis of SMs.^73^ Future studies using combination of ZnONPs along with humic acid treatment to evaluate if this later could be a solution to subside the toxic effects of ZnONPs would be of particular interest.

#### 3.2.3. Elicitation potential of iron oxide nanoparticles (Fe_3_O_4_NPs)

Their simple and cost-effective synthesis makes iron oxide nanoparticles (Fe_3_O_4_NPs) readily available for various applications in various fields of science including their potential use as elicitors of plant SMs production.^20,81–83^ For instance, Fe_3_O_4_NPs can be more effective than other MONPs such as ZnONPs to elicit SMs production, as suggested with hairy root culture of *Cichorium intybus*. Indeed, results showed that contrary to ZnONPs, Fe_2_O_3_NPs application proved to be proficient elicitor treatment to enhance both growth and production of phenolic (4.65 mg g^−1^ DW) and flavonoid (77.34 μg g^−1^ DW) compounds in hairy root culture of *C. intybus*.^82^ Another interesting example of the use of Fe_3_O_4_NPs was obtained with *Hypericum perforatum*. This plant is one of top selling product in industrial based countries for medicinal uses, due to its accumulation of particular SMs: hypericin (3-fold) and hyperforin (13-fold). *H. perforatum in vitro* cultures have attracted an active search, including the evaluation of a large number of different elicitors, in order to enhance their production at acceptable levels for commercial applications.^84,85^ Interestingly, in a study was conducted with cell suspension cultures of *H. perforatum*, after testing several concentrations, Fe_3_O_4_NPs (100 ppb) showed their ability to specifically increase the production of hyperforin as compared to the hypericin.^83^ These results suggested, following optimization, the possibility of directing a metabolic pathway toward the synthesis of the desired compound. Another study evidenced the synergistic effect of foliar application of Fe_3_O_4_NPs on Cress (*Lepidum sativum* L.) plants resulting from gamma-irradiated seeds.^81^ Fe_3_O_4_NPs (30 ppb) significantly increased the production of both total phenolic (13027 mg GAE per g), and flavonoid (453 mg QE per g) contents, and a synergistic effect with a prior gamma ray treatment of the seeds was noted. Moreover, repeated elicitation with Fe_3_O_4_NPs was able to further potentiate the production of these SMs.^81^ Nevertheless, although all these results are very promising, a prior optimization of Fe_3_O_4_NPs concentrations and treatment duration to applied are nevertheless necessary to avoid undesirable toxic effects, as indicated in the latest study conducted on hairy root culture of *Hyoscyamus reticulatus*. Applied at different concentrations (0, 450, 900, 1800, and 3600 mg L^−1^) and for different exposure times (24, 48, and 72 h), results showed a concentration- and time-dependent activation of the tropane alkaloids (5-fold; hyoscyamine and scopolamine) biosynthesis, in 450 and 900 mg L^−1^ of Fe_3_O_4_NPs-treated cultures after 24 h and 48 h of exposure time, respectively. However, the prolonged exposure time (*i.e.* 96 h) led to decrease in tropane alkaloids (2-fold) production and suggested potential toxic effects of Fe_3_O_4_NPs.^20^

#### 3.2.4. Elicitation potential of titanium dioxide nanoparticles (TiO_2_NPs)

Titanium dioxide nanoparticles (TiO_2_NPs) are one of the most widely released nanoparticles in the environment, especially because of their wide use as a UV filter in sunscreens. Therefore, plants are more prone to TiO_2_NPs they can directly uptake from environment.^86^ The most recent studies suggested that the physiological effects of TiO_2_NPs on plants are mainly caused by ionic Ti.^87,88^ Various research groups have also investigated the elicitation potential of TiO_2_NPs on plant SMs production using both *in vivo* and *in vitro* systems.^89–91^ Using *in vitro* systems, aloin (118%) content was increased in cell suspension culture of *Aloe vera* when treated with TiO_2_NPs.^35^ Similarly, the phenolic and flavonoid contents in callus culture of *Cicer arietinum* were increased when exposed to different concentrations of TiO_2_NPs.^89^ The authors reported a significant dose-dependent increase in the production of gallic acid, *o*-coumaric acid and tannic acid in response to 6 mg L^−1^ TiO_2_NPs, whereas the chlorogenic acid and *t*-cinnamic acid at 4.5 mg L^−1^ TiO_2_NPs. Using *in vivo* systems, application of different concentrations (0, 10, 50, 100, 200 and 1000 mg L^−1^) of TiO_2_NPs (10–15 nm size) on seedlings of *Salvia officinalis* aslo evidenced a dose-dependent response with highest accumulation of total phenolic content (35.2 mg g^−1^ DW) at a 200 mg L^−1^ dose, while the highest total flavonoids content (21.9 mg g^−1^ DW) at 100 mg L^−1^ treatment of TiO_2_NPs was observed. Moreover, a significant increase in essential oil content at a dose of 200 mg L^−1^ TiO_2_NPs was also noted.^90^ A study conducted on *Hyoscyamus niger*, compared the impact of TiO_2_NPs *vs.* bulk titanium, showed a significant biomass (dry weight) improvement for plants treated with 40 mg L^−1^ of TiO_2_NPs (10–15 nm size), whereas bulk titanium was ineffective on this growth parameter. On the SMs production, highest content of hyoscyamine (0.286 g kg^−1^) was observed with a concentration of 80 mg L^−1^ TiO_2_NPs, while highest scopolamine content (126 g kg^−1^) was reported at a lower concentration of TiO_2_NPs (20 mg L^−1^).^91^ In a recent study, the complex effect of drought stress and different concentrations TiO_2_NPs (0, 10 and 40 ppm) on dragonhead *Dracocephalum moldavica* was reported by^92^ in a factorial experiment. Under normal irrigation, foliar application of TiO_2_NPs at 10 ppm improved plant biomass as well as essential oils production. Furthermore, the Authors showed that drought stress-induced oxidative damages can be overpassed by foliar application of TiO_2_NPs at appropriate concentrations. This result is of particular interest in the context of increasing temperatures due to climate change and should deserve additional research considerations.

#### 3.2.5. Elicitation potential of cerium oxide nanoparticles (CeO_2_NPs)

Cerium oxide nanoparticles (CeO_2_NPs) have been found to be very stable with limited dissolution in soil, however, root exudates could enhance the solubility of CeO_2_NPs, leading to significant accumulation of Ce in plant tissues.^93,94^ Cerium (Ce) is a rare earth elements (REEs), and although it is not essential for plants, it have been reported to stimulate growth and other physiological processes.^95^ There are also many reports are present that demonstrate the potential impact of CeO_2_NPs on plant primary metabolism, but a few literature on their impact(s) on plant SMs production is so far exiting.^95–97^ Hydroponic culture of *Solanum lycopersicum* elicited with CeO_2_NPs (0.1 Mm, 33.05 nm size) showed a significant increase in their carotenoid contents (26%).^96^ Comparatively, both applications of CeO_2_NPs (1000 mg L^−1^) and indium oxide NPs (In_2_O_3_NPs, 250 mg L^−1^) induced an oxidative stress associated with an increase in production of phenolic compounds (27%) in plantlets of *A. thaliana*.^97^ On the contrary, application of CeO_2_NPs has been shown to improve the radical scavenging potency upto 32% of radish (*Raphanus sativus*) seedlings but have no effect on the production of phenolic and flavonoid compounds.^95^

#### 3.2.6. Elicitation potential of aluminum (Al_2_O_3_NPs), manganese (Mn_2_O_3_NPs) and cadmium (CdONPs) oxide nanoparticles

Aluminium oxide (Al_2_O_3_), cadmium oxide (CdO) and manganese dioxide (Mn_2_O_3_) nanoparticles are to date the least exploited nanomaterials as elicitors of plant SMs. Application of Al_2_O_3_NPs on tobacco BY-2 cell suspension culture led to substantial increase in phenolic content (62%) in a dose-dependent way. A stimulation of biomass production (fresh weight) was also observed at 100 μg mL^−1^ Al_2_O_3_NPs.^98^ A study with *Hordeum vulgare* (barley) plants exposed to CdONPs (ranging from 7–60 nm in size and 2.03 × 10^5^ particles per cm^3^ concentration) reported an increased production of isovitexin (183%) and ferulic acid (30%) and also suggested a rapid penetration of CdONPs through soil along with water into the plants as compared to foliar spray.^99^ A very little is known about the interaction of plants with Mn_2_O_3_NPs. A study was conducted to explore the prospective effects of Mn_2_O_3_NPs on morphology, physiology and specialized metabolism in shoot tip cultures of *Atropa belladonna*. Results showed that, at appropriate dosage (25 mg L^−1^), Mn_2_O_3_NPs were found to stimulate both plant growth and SMs production (23% increase in alkaloids, 12% in total phenolic and 32% in flavonoid contents). From this study, Mn_2_O_3_NPs would be further considered as a novel extracellular elicitor of SMs in *in vitro* cultures of medicinally or biotechnologically important plant species.^100^

#### 3.2.7. Elicitation potential of silicon dioxide nanoparticles (SiO_2_NPs)

To date, only two studies have been carried out to investigate the effect of silicon dioxide nanoparticles (SiO_2_NPs) on specialized metabolism of plants. The first study, reported a 2-fold increase in biosynthesis of rosmarinic acid, xanthomicrol, isokaempferide and cirsimaritin in the hairy root culture of *Dracocephalum kotschyi*, after 48 h of exposure to SiO_2_NPs (100 nm).^101^ The second report, in early flowering plants of *Nigella sativa* TiO_2_NPs were found to be more effective than SiO_2_NPs in stimulating the production of thymoquinone.^102^

### 3.3. Carbon-related nanomaterials as elicitor of specialized metabolites of plants

Beside MNPs and MONPs, the other nanomaterials include semiconductor quantum dots, polymeric nanoparticles, dendrimers and carbon based nanomaterials. Among these other nanomaterials, carbon-based nanomaterials were widely used in agriculture biotechnology to check their effects on primary and specialized metabolisms of various plant species.^74,103,104^ Carbon based nanomaterials include, single-walled carbon nanotubes (SWCNTs), multi-walled carbon nanotubes (MWCNTs), and derivatives of fullerene (Buckminsterfullerene or Buckyball). Literature data showed that SWCNTs and MWCNTs affect the physiology, growth and metabolism of plants in diverse ways.^105,106^ Besides these, different studies suggested that the carbon nanotubes and fullerenes could potentially modify the expression level of genes involved in plant specialized metabolism and therefore can act as potent elicitors of various commercially important SMs.^107–109^Table 3 summarize the elicitation potentials in various plant species of carbon based nanomaterials as well as of chitosan-NPs also discussed in the present section.

**Table 3:** Summary of the effects of different types of carbon-related nanomaterials used as elicitors of specialized metabolites in different plant species

NPs	Size of NPs (nm)	Effective concentration of NPs	Plant species	Culture type	Growth media/conditions	Elicitation of specialized metabolites	Phytotoxicity	References
SWCNTs	110–170	0.002 g L^−1^	*Simmondsia chinensis*	Plantlets	MS media	Phenolic (23.17 mg GAE per g DW), flavonoids (20.66 mg QE per g DW) and tannins (6.35 mg TE per g DW) contents were increased	—	108
SWCNTs	—	500 mg L^−1^	*Tanacetum parthenium*	Whole plant	Green house	Parthenolide contents (2-fold) were increased	—	107
MWCNTs	—	100 μg mL^−1^	*Satureja khuzestanica*.	Callus	B5 basal media	Total contents of phenolic (12%), flavonoids (3%), rosmarinic acid (12.32 mg g^−1^ DW) and caffeic acid (9.2 mg g^−1^ DW) were increased	—	109
Fullerene	1.5–5.0	10.8 mM	*Momordica charantia*	Seed germination	3B potting mix	Increase in production of anticancerous (cucurbitacin-B, 74% and lycopene, 82%) and antidiabetic (charantin, 20% and insulin, 90%) compounds	—	110
Chitosan-NP	40–180	0.01%	*Camellia sinensis*	Foliar spray on leaves	Hydroponic	20% increase in accumulation of phenolic and 24% in flavonoid compounds	—	111

#### 3.3.1. Elicitation potential of carbon nanotubes

Concerning SWCNTs,^107^ evaluating the impact of different concentrations (125, 150, 250 and 500 mg L^−1^) on whole plant culture of *Tanacetum parthenium* showed a favorable effect on the accumulation of parthenolide contents (2.5-fold) in shoot treated at a 500 mg L^−1^ SWCNTs concentration level.^107^ On *in vitro* cultures, a significant increase in total phenolic, flavonoid and tannin contents in shoot culture of *Simmondsia chinensis* treated with SWCNTs (0.002 g L^−1^) was reported.^108^

The inductive effect of MWCNTs on SMs production was also reported on *in vitro* callus cultures of *Satureja khuzestanica* with an optimal concentration of 100 μg mL^−1^ MWCNTs, improving both biomass and accumulation of total phenolic (12%), flavonoids (3%), rosmarinic acid (12.32 mg g^−1^ DW) and caffeic acid (9.2 mg g^−1^ DW) contents.^109^

Kole *et al.*,^110^ have studied the effect of fullerenes (10.8 Mm) on seed germination of bitter melon (*M. charantia*) and showed a significant increase in seedling growth rate and in anticancer (cucurbitacin-B, 74% and lycopene, 82%) and antidiabetic (charantin, 20% and insulin, 90%) compounds accumulation after fullerenes exposure.

#### 3.3.2. Elicitation potential of chitosan nanoparticles

Chitosan is widely used for agriculture and biomedicine purposes. It possess attractive unique properties like its non-toxicity and biodegradability. To date, only one report is available on the elicitation behavior of chitosan-NPs (40–180 nm) used as potent elicitors of gallic acid, epicatechin, epigallocatechin, epigallocatechin-gallate and caffeine accumulation in tea (*Camellia sinensis*) plants growing under hydroponic conditions.^111^

## 4. Postulated mechanism of elicitation of specialized metabolites by nanomaterials

**Fig. 4:**
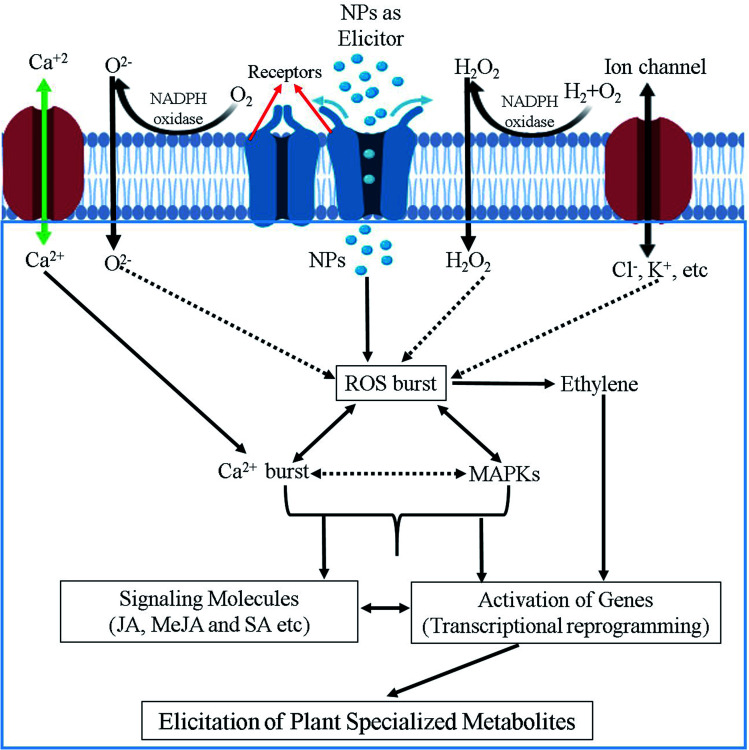
Schematic illustration of the possible mechanism involved in NPs-mediated elicitation of specialized metabolites in plants.

Although a number of reports suggested various signaling pathways to explain the modulation capacity of nanomaterials on the production of plant SMs, to date the precise mechanism still remains elusive.^10,51,84,109^ Probably, because elicitation mechanisms are very complex, and could involve thousands of mediators from multiple signaling pathways and their crosstalk. Additionally, all these events fluctuate depending on the origin, specificity, exposure type, time and concentration of elicitors along with the plant dependent parameter such as the plant species developmental stage, cellular cycle, type of tissue, nutritional conditions, elicitor uptake by media/soil/aerial, physiochemical environment and so on. It is, therefore, very challenging to propose a universal model for the elicitation mechanism of plant SMs production triggered by nanomaterials. We anticipate that the initial responses of plants to NPs might include calcium ion (Ca^2+^) and Ca^2+^ flux movements and ROS produced by oxidative burst as important second messengers leading to the (up) regulation/phosphorylation of mitogen-activated protein kinase (MAPK) cascades regulation the transcriptional levels of master regulators of plant SMs biosynthesis, which are a common feature observed for many abiotic elicitors. Fig. 4 illustrate a hypothesized mechanism for the elicitation of plant SMs by nanomaterials summarizing the view of different researchers.^10,11,112^ NPs mostly act as elicitation signal that could interact with elicitor binding sites and/or receptors present at the surface of the plant cell membrane. Upon recognition, interaction and/or binding phase(s), a cascade of events are activated as mentioned above. The initial plant response to NPs is most probably involve an active exchange of ions, for instance Na^+^/K^+^/Cl^−^ effluxes and Ca^2+^/H^+^ influxes through plasma membrane into the cytosol. Ca^2+^ influx is considered as the most important event because of its diverse involvement in various physiological and cellular pathways,^113^ and can probably play a pivotal role in the first steps of this elicitation mechanism. ROS generation in response to NPs is another important and widely proposed event that can be implied in the elicitation process of plant SMs production. NADPH oxidase and other related oxidases, sometime activated through differential Ca^2+^ movements, are responsible for generation of ROS in plant cells.^114–116^ This oxidative burst in plant cell can result in the activation of cGMP-dependent protein kinase. These activated protein kinases cause the phosphorylation of mitogen-activated protein kinases (MAPKs), which in turn results in gene expression modulation events. All these transcriptional reprogramming events can further activate the pathways of SMs production in plant cells.^117^ G-proteins (aka guanine nucleotide-binding proteins) can also activate the SMs accumulation indirectly through the *de novo* biosynthesis of stress signaling compounds such as salicyclic acid (SA), jasmonic acid (JA) and methyl jasmonic acid (MeJA), facilitating plant defense response through the reprogramming of plant specialized metabolism pathways (Fig. 4).^118^

## 5. Negative impact of nanomaterials to plant cells and their metabolism

**Fig. 5:**
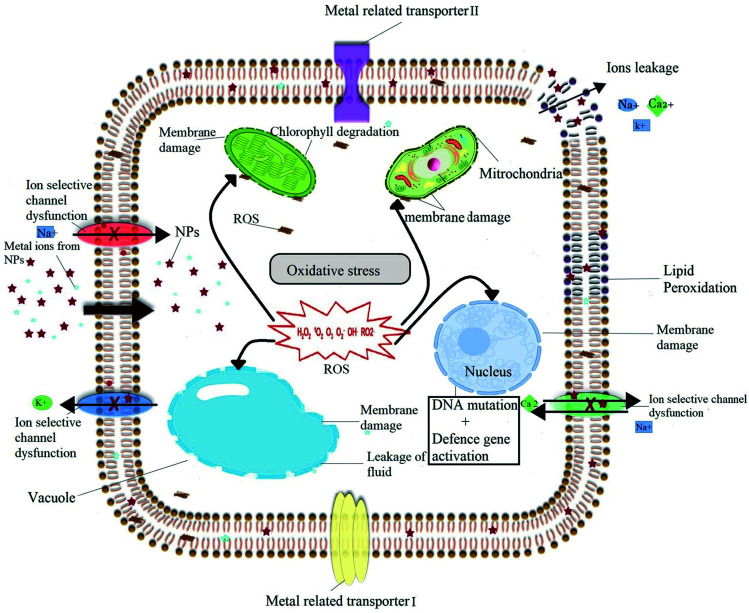
Diagram showing the possible phytotoxicity induced by the NPs through generation of excessive amount of ROS that could damage nuclear material and cell membranes of various organelles that can eventually lead to cell death.

Despite of numerous advantages, nanomaterials present also some adverse effects on plant physiology, biochemistry, primary and specialized metabolism under certain conditions. All the notorious effects caused by nanomaterials to plant come under the heading of phytotoxicity. Phytotoxicity of nanomaterials depends on a broad range of factors including the type, age, growth medium and growing conditions of the considered plant species along with the nature, exposure time and physicochemical characteristics of nanomaterials used. The exact mechanism of phytotoxicity of nanomaterials is still unclear, but many hypotheses have been proposed. The detailed of proposed phytotoxicity mechanism of nanomaterials is illustrated in Fig. 5. Most commonly proposed mechanism of phytotoxicity of nanomaterials is related with the induced oxidative burst and the generation of ROS, which play a critical role in determining the nature and the type of phytotoxicity.^21,119,120^ ROS can cause oxidative stress, which emerges when the ROS level exceeds the defense mechanisms, and is able to damage plant cells by inducing DNA damage,^121^ membrane damage,^122^ protein oxidation,^123^ lipid peroxidation^124^ and/or electrolyte leakage finally leading to cell death.^125^ In the present review we have already discussed both the positive impact of nanomaterials on the production of plant SMs and biomass, but also presented some examples of negative impact of nanomaterials, growth, viability and/or on primary and specialized metabolism in various plant species (in particular see ref. 42, 68 and 69).

Details of available literature on any phytotoxicity caused by NPs is also given in Tables 1, 2 and 3.

To summarize the available literature, a consensus exists on the application of high concentration of nanomaterials.^20,80,92^ This was, for example, illustrated by^40^ with plantlets of *C. officinalis* exposed to AgNPs at 400 mg L^−1^. Similarly, phytotoxic effects were observed on seedling of *S. melongena*^80^ treated with 1000 mg L^−1^ CuO, ZnO or NiO NPs. Primary metabolism and plant growth can be also affected, as observed with nano-zerovalent iron presenting inhibitory effects on the chlorophyll and carotenoid content of rice when used in higher concentration, which in turn decrease the rate of photosynthesis and plant biomass accumulation.^126^ Cell viability could be directly impacted by excessive ROS generation as observed for MWCNTs treated-cell suspension culture of rice.^127^

From the literature review of different studies, it has been evident to us that the smaller size and excess of nanomaterials as well as long exposure time could be harmful for plants and might be a cause of phytotoxicity while used under optimized conditions nanomaterials may be beneficial for plant growth and SMs production. Optimizing these conditions for each plant species and culture system is therefore a prerequisite to investigate carefully when the use of nanomaterials as elicitor of plant SMs production is considered.

## 6. Conclusions and future outlooks

The current review suggests that nanomaterials (MNPs, MONPs and other carbon related nanomaterials) are promising tools to improve the quality and quantity of valuable plant SMs in different culture systems. Among different abiotic elicitors used to date, nanomaterials emerge as efficient elicitors of plant SMs production both in terms of their specificity and productivity. Indeed, enhanced production of a large number of commercially important SMs have been reported in various plant species grown under different culture conditions by using different types of nanomaterials. These results pave the way of a more systematic consideration of nanomaterials as elicitors in plant science.

Overall critical analysis of all reported data on elicitation of plant SMs by nanomaterials showed that the AgNPs and ZnONPs (among all other NPs) proved to be more efficient elicitors both in terms of their productivity and specificity. For production of plant SMs at commercial levels, different researchers reported different optimum concentration of nanomaterials depending on type of culture and plant species used. Most of the published data reported that the lower concentrations of nanomaterials are more efficient in eliciting plant SMs than higher ones, probably due to toxic effects of nanomaterials to plant primary metabolism.

Researches performed on the elicitation of SMs by different types of nanomaterials have demonstrated that the elicitation mechanism is complex and depends on various factors such as the size, morphology, concentration and exposure time of NPs along with the considered plant species and the type of the growing conditions, resulting in different consequences.^127–130^ Paucity of knowledge on the different factors affecting elicitation mechanism by nanomaterials is aggravated by the fact that most of the studies focused on plant growth, development, physiological, including primary metabolism, parameters, but the effect of nanomaterials on plant SMs production is scarcely studied and many questions remain unexplored. For example, an interesting and promising future line of research also includes studying the antagonistic or synergistic effects of one class of nanomaterials on specialized metabolism in combination with another class of nanomaterials or with other abiotic/biotic elicitors.

Lack of unanimity exists on the mechanism of elicitation by nanomaterials, as the proposed mechanism may vary according to the plant species, culture conditions and/or type of nanomaterials used. In addition, application routes of the nanomaterials and therefore different mode of exposure, entry and/or translocation into the plant cells and tissues, internalization could lead to different mode of action.^10,41,72,103,131,132^ Plant specialized metabolism includes a wide range of compounds and their biosynthesis are tightly controlled by signaling events. The impact on plant SMs production differs according their phytochemical classes and plant species. A case-by-case analysis of NP type and plant species may be required to better understand the elicitation mechanism. For instance, it would be interesting to consider the accumulation of a particular class of SMs produced in the same plant species grown under in different culture conditions and systems (field or greenhouse *vs.* different *in vitro* systems). It is, therefore very difficult to propose a general elicitation mechanism for nanomaterials, and their impact on plant SMs production and more studies are required for its comprehensive understanding. Omics-based analyses (*e.g.* genomics, transcriptomics, proteomics and metabolomics) have to be more systematically considered in order to identify the molecular mechanisms of plant SMs elicitation resulting from nanomaterials applications.

The open challenges for biotechnologists and nanotechnologists to continue their research in this area are: to determine the critical quantities of nanomaterials that plants can safely absorb without showing any signs of phytotoxicity and to understand the mechanistic interactions between the nanomaterials and the plant cells and their SMs pathways. In particular, further researches focusing on the comprehensive identification of the cellular and molecular events responsible for the observed phytotoxic effects of nanomaterials are strongly needed to take a full advantage of the stimulation capacities of these new classes of elicitors on plant SMs production prior to future applications.

Moreover, till now only a few types of nanomaterials are explored as potent elicitors of plant SMs (Ag, Au, Zn, ZnO, Cu, CuO, CdO, Al_2_O_3_, CeO_2_, SiO_2_, Ni and MgO) whereas, the role of a large number of nanomaterials (Fe_2_O_3_, Co, CoO, NiO, Nd_2_O_3_, fullerene, fullerols, graphene, GO and carbon dots) remain unexplored. These nanomaterials can also act as novel elicitors of plant SMs, and further research should be carried out to find out their possible role in elicitation of valuable secondary metabolites of plants.

## Funding source

This research did not receive any specific grant from funding agencies in the public, commercial, or not-for-profit sectors.

## Conflicts of interest

There are no conflicts to declare.

## Supplementary Material

## References

[cit1] Sudha P. N. , Sangeetha K., Vijayalakshmi K. and Barhoum A., in Emerging Applications of Nanoparticles and Architecture Nanostructures, Elsevier, 2018, pp. 341–384

[cit2] Ye F., Zhao Y., El-Sayed R., Muhammed M., Hassan M. (2018). Nano Today.

[cit3] Xia Y. (2014). Angew. Chem., Int. Ed..

[cit4] Cardoso V. F., Francesko A., Ribeiro C., Bañobre-López M., Martins P., Lanceros-Mendez S. (2018). Adv. Healthcare Mater..

[cit5] Kim D., Shin K., Kwon S. G., Hyeon T. (2018). Adv. Mater..

[cit6] Chhipa H. (2017). Environ. Chem. Lett..

[cit7] Duhan J. S., Kumar R., Kumar N., Kaur P., Nehra K., Duhan S. (2017). Biotechnology Reports.

[cit8] Servin A., Elmer W., Mukherjee A., De la Torre-Roche R., Hamdi H., White J. C., Bindraban P., Dimkpa C. (2015). J. Nanopart. Res..

[cit9] Feizi H., Amirmoradi S., Abdollahi F., Pour S. J. (2013). Annu. Res. Rev. Biol..

[cit10] Hatami M., Kariman K., Ghorbanpour M. (2016). Sci. Total Environ..

[cit11] Marslin G., Sheeba C. J., Franklin G. (2017). Frontiers in Plant Science.

[cit12] Baikar S., Malpathak N. (2010). Pharmacogn. Rev..

[cit13] Greenwell M., Rahman P. (2015). Int. J. Pharm. Sci. Res..

[cit14] Seca A., Pinto D. (2018). Int. J. Mol. Sci..

[cit15] Ali M., Abbasi B. H., Ahmad N., Khan H., Ali G. S. (2017). Crit. Rev. Biotechnol..

[cit16] Giri C. C., Zaheer M. (2016). Plant Cell, Tissue Organ Cult..

[cit17] Hussain M. S., Fareed S., Saba Ansari M., Rahman A., Ahmad I. Z., Saeed M. (2012). J. Pharm. BioAllied Sci..

[cit18] Asl K. R., Hosseini B., Sharafi A., Palazon J. (2019). Eng. Life Sci..

[cit19] Bhat P., Bhat A. (2016). J. Exp. Sci..

[cit20] Moharrami F., Hosseini B., Sharafi A., Farjaminezhad M. (2017). In Vitro Cell. Dev. Biol.: Plant.

[cit21] Ma C., White J. C., Dhankher O. P., Xing B. (2015). Environ. Sci. Technol..

[cit22] Marchiol L., Mattiello A., Pošćić F., Giordano C., Musetti R. (2014). Nanoscale Res. Lett..

[cit23] Wang P., Lombi E., Zhao F.-J., Kopittke P. M. (2016). Trends Plant Sci..

[cit24] Aslani F., Bagheri S., Muhd Julkapli N., Juraimi A. S., Hashemi F. S. G., Baghdadi A. (2014). Sci. World J..

[cit25] Nair R., Varghese S. H., Nair B. G., Maekawa T., Yoshida Y., Kumar D. S. (2010). Plant Sci..

[cit26] Riaza M. S. , Ullaha N., Alid H. and Nadhmane A., Analysis, Fate, and Toxicity of Engineered Nanomaterials in Plants, 2019, vol. 84, p. 23

[cit27] Deng Y.-q., White J. C., Xing B.-s. (2014). J. Zhejiang Univ., Sci., A.

[cit28] Chung I.-M., Rekha K., Rajakumar G., Thiruvengadam M. (2018). 3 Biotech.

[cit29] Chung I.-M., Rekha K., Rajakumar G., Thiruvengadam M. (2018). Bioprocess Biosyst. Eng..

[cit30] Golkar P., Moradi M., Garousi G. A. (2018). Sugar Tech.

[cit31] Chung I.-M., Rajakumar G., Thiruvengadam M. (2018). Acta Biol. Hung..

[cit32] Gupta S. D., Agarwal A., Pradhan S. (2018). Ecotoxicol. Environ. Saf..

[cit33] Yasur J., Rani P. U. (2013). Environ. Sci. Pollut. Res..

[cit34] Zahir A., Nadeem M., Ahmad W., Giglioli-Guivarc'h N., Hano C., Abbasi B. H. (2019). Plant Cell, Tissue Organ Cult..

[cit35] Raei M., Angaji S. A., Omidi M., Khodayari M. (2014). Int. J. Biosci..

[cit36] Shakeran Z., Keyhanfar M., Asghari G., Ghanadian M. (2015). Turk. J. Biol..

[cit37] Zhang B., Zheng L. P., Yi Li W., Wen Wang J. (2013). Curr. Nanosci..

[cit38] Ghasemi B., Hosseini R., Nayeri F. D. (2015). Turk. J. Bot..

[cit39] Tahoori F., Ahmad M., Nejadsattari T., Ofoghi H., Iranbakhsh A. (2019). Not. Bot. Horti Agrobot. Cluj-Napoca.

[cit40] Ghanati F., Bakhtiarian S. (2014). Trop. J. Pharm. Res..

[cit41] Jamshidi M., Ghanati F. (2017). Plant Physiol. Biochem..

[cit42] Jamshidi M., Ghanati F., Rezaei A., Bemani E. (2016). Cytotechnology.

[cit43] Aghajani Z., Ekhtiyari R. (2013). Afr. J. Agric. Res..

[cit44] Abbasi Khalaki M., Ghorbani A., Moameri M. (2016). Journal of Rangeland Science.

[cit45] Azeez L., Lateef A., Adebisi S. A. (2017). Appl. Nanosci..

[cit46] Jasim B., Thomas R., Mathew J., Radhakrishnan E. (2017). Saudi Pharm. J..

[cit47] Syu Y.-y., Hung J.-H., Chen J.-C., Chuang H.-w. (2014). Plant Physiol. Biochem..

[cit48] Spinoso-Castillo J., Chavez-Santoscoy R., Bogdanchikova N., Pérez-Sato J., Morales-Ramos V., Bello-Bello J. (2017). Plant Cell, Tissue Organ Cult..

[cit49] García-Sánchez S., Bernales I., Cristobal S. (2015). BMC Genomics.

[cit50] Logeswari P., Silambarasan S., Abraham J. (2015). J. Saudi Chem. Soc..

[cit51] Ali A., Mohammad S., Khan M. A., Raja N. I., Arif M., Kamil A., Mashwani Z.-u.-R. (2019). Artif. Cells, Nanomed., Biotechnol..

[cit52] Golkar P., Moradi M., Garousi G. A. (2019). Sugar Tech.

[cit53] Krishnaraj C., Jagan E., Ramachandran R., Abirami S., Mohan N., Kalaichelvan P. (2012). Process Biochem..

[cit54] Genady E. A., Qaid E. A., Fahmy A. H. (2016). Int. J. Pharm. Res. Allied Sci..

[cit55] López-Vargas E., Ortega-Ortíz H., Cadenas-Pliego G., de Alba Romenus K., Cabrera de la Fuente M., Benavides-Mendoza A., Juárez-Maldonado A. (2018). Appl. Sci..

[cit56] Talankova-Sereda T. , Liapina K., Shkopinskij E., Ustinov A., Kovalyova A., Dulnev P. and Kucenko N., in Nanophysics, Nanophotonics, Surface Studies, and Applications, Springer, 2016, pp. 427–436

[cit57] Zhao L., Huang Y., Hu J., Zhou H., Adeleye A. S., Keller A. A. (2016). Environ. Sci. Technol..

[cit58] Fazal H., Abbasi B. H., Ahmad N., Ali M. (2016). Appl. Biochem. Biotechnol..

[cit59] Ghazal B., Saif S., Farid K., Khan A., Rehman S., Reshma A., Fazal H., Ali M., Ahmad A., Rahman L. (2018). IET Nanobiotechnol..

[cit60] Singh R., Singh D. P., Gupta P., Jain P., Mishra T., Kumar A., Dhawan S. S., Shirke P. A. (2019). Ind. Crops Prod..

[cit61] Fazal H., Abbasi B. H., Ahmad N., Ali M., Shujait Ali S., Khan A., Wei D.-Q. (2019). Artif. Cells, Nanomed., Biotechnol..

[cit62] Wesołowska A., Jadczak P., Kulpa D., Przewodowski W. (2019). Molecules.

[cit63] Chung I.-M., Rekha K., Venkidasamy B., Thiruvengadam M. (2019). Water, Air, Soil Pollut..

[cit64] Chung I.-M., Thiruvengadam M. (2019). Appl. Sci..

[cit65] Khan M. A., Khan T., Riaz M. S., Ullah N., Ali H., Nadhman A. (2019). Adv. Colloid Interface Sci..

[cit66] Zuverza-Mena N., Martinez-Fernandez D., Du W., Hernandez-Viezcas J. A., Bonilla-Bird N., Lopez-Moreno M. L., Komarek M., Peralta-Videa J. R., Gardea-Torresdey J. L. (2017). Plant Physiol. Biochem..

[cit67] Rajput V., Minkina T., Suskova S., Mandzhieva S., Tsitsuashvili V., Chapligin V., Fedorenko A. (2018). J. Bionanosci..

[cit68] Yruela I. (2005). Braz. J. Plant Physiol..

[cit69] ul Ain N., ul Haq I., Abbasi B. H., Javed R., Zia M. (2017). IET Nanobiotechnol..

[cit70] Singh O. S., Pant N. C., Laishram M., Tewari R. D., Joshi K., Pandey C. (2018). J. Pharmacogn. Phytochem..

[cit71] Chung I.-M., Rekha K., Rajakumar G., Thiruvengadam M. (2016). 3 Biotech.

[cit72] Laishram L., Pant N. C., Singh O. S., Dhoundiyal R., Joshi K., Pandey C. (2018). Int. J. Conserv. Sci..

[cit73] Chamani E., Karimi Ghalehtaki S., Mohebodini M., Ghanbari A. (2015). Iranian Journal of Genetics and Plant Breeding.

[cit74] Kołodziejczak-Radzimska A., Jesionowski T. (2014). Materials.

[cit75] Oloumi H., Soltaninejad R., Baghizadeh A. (2015). Indian J. Plant Physiol..

[cit76] Karimi N., Behbahani M., Dini G., Razmjou A. (2018). Adv. Nat. Sci.: Nanosci. Nanotechnol..

[cit77] Raigond P., Raigond B., Kaundal B., Singh B., Joshi A., Dutt S. (2017). J. Environ. Biol..

[cit78] Javed R., Usman M., Yücesan B., Zia M., Gürel E. (2017). Plant Physiol. Biochem..

[cit79] Javed R., Yucesan B., Zia M., Gurel E. (2018). Sugar Tech.

[cit80] Baskar V., Nayeem S., Kuppuraj S. P., Muthu T., Ramalingam S. (2018). 3 Biotech.

[cit81] Ahamed T. E. S. (2018). J. Plant Sci..

[cit82] Mohebodini M., Fathi R., Mehri N. (2017). Iranian Journal of Genetics and Plant Breeding.

[cit83] Sharafi E., Khayam Nekoei S., Fotokian M. H., Davoodi D., Hadavand Mirzaei H., Hasanloo T. (2013). Journal for the Measurement of Physical Behaviour.

[cit84] Shakya P., Marslin G., Siram K., Beerhues L., Franklin G. (2019). J. Pharm. Pharmacol..

[cit85] Wang J., Qian J., Yao L., Lu Y. (2015). Bioresources and Bioprocessing.

[cit86] Keller A. A., McFerran S., Lazareva A., Suh S. (2013). J. Nanopart. Res..

[cit87] Antisari L. V., Carbone S., Gatti A., Vianello G., Nannipieri P. (2015). Environ. Sci. Pollut. Res..

[cit88] Lei Z., Mingyu S., Xiao W., Chao L., Chunxiang Q., Liang C., Hao H., Xiaoqing L., Fashui H. (2008). Biol. Trace Elem. Res..

[cit89] AL-oubaidi H. K. M., Kasid N. M. (2015). World J. Pharm. Res..

[cit90] Ghorbanpour M. (2015). Indian J. Plant Physiol..

[cit91] Ghorbanpour M., Hatami M., Hatami M. (2015). Acta Agric. Slov..

[cit92] Kamalizadeh M., Bihamta M., Zarei A. (2019). Acta Physiol. Plant..

[cit93] Rui Y., Zhang P., Zhang Y., Ma Y., He X., Gui X., Li Y., Zhang J., Zheng L., Chu S. (2015). Environ. Pollut..

[cit94] Schwabe F., Tanner S., Schulin R., Rotzetter A., Stark W., Von Quadt A., Nowack B. (2015). Metallomics.

[cit95] Corral-Diaz B., Peralta-Videa J. R., Alvarez-Parrilla E., Rodrigo-García J., Morales M. I., Osuna-Avila P., Niu G., Hernandez-Viezcas J. A., Gardea-Torresdey J. L. (2014). Plant Physiol. Biochem..

[cit96] Hussain I., Singh N., Singh A., Singh H., Singh S., Yadav V. (2017). Sci. Hortic..

[cit97] Nelson B. C., Coskun S. H., Ma C., Li H., Guo H., Musante C., White J. C., Xing B., Dhankher O. P. (2016). Environ. Sci.: Nano.

[cit98] Poborilova Z., Opatrilova R., Babula P. (2013). Environ. Exp. Bot..

[cit99] Pompeiano A. , Ãska J. T., Oravec M. and Urban O., Environmental pollution, 2016 10.1016/j.envpol.2016.05.013 27503055

[cit100] Tian H., Ghorbanpour M., Kariman K. (2018). Ind. Crops Prod..

[cit101] Nourozi E., Hosseini B., Maleki R., Mandoulakani B. A. (2019). Ind. Crops Prod..

[cit102] Kahila M. M. H., Najy A. M., Rahaie M., Mir-Derikvand M. (2018). Nat. Prod. Res..

[cit103] Husen A., Siddiqi K. S. (2014). J. Nanobiotechnol..

[cit104] Ma X., Geiser-Lee J., Deng Y., Kolmakov A. (2010). Sci. Total Environ..

[cit105] Lin S., Reppert J., Hu Q., Hudson J. S., Reid M. L., Ratnikova T. A., Rao A. M., Luo H., Ke P. C. (2009). Small.

[cit106] Miralles P., Johnson E., Church T. L., Harris A. T. (2012). J. R. Soc., Interface.

[cit107] Ahmadi S., Ghorbanpour M., Hadian J., Salehi-Arjmand H. (2018). Journal of Medicinal Plants.

[cit108] Gaafar A. A., Taha R. A., Abou-Baker N. H., Shaaban E. A., Salama Z. A. (2018). Biosci. Res..

[cit109] Ghorbanpour M., Hadian J. (2015). Carbon.

[cit110] Kole C., Kole P., Randunu K. M., Choudhary P., Podila R., Ke P. C., Rao A. M., Marcus R. K. (2013). BMC Biotechnol..

[cit111] Chandra S., Chakraborty N., Dasgupta A., Sarkar J., Panda K., Acharya K. (2015). Sci. Rep..

[cit112] Sosan A., Svistunenko D., Straltsova D., Tsiurkina K., Smolich I., Lawson T., Subramaniam S., Golovko V., Anderson D., Sokolik A. (2016). Plant J..

[cit113] Bolwell G. P., Wojtaszek P. (1997). Physiol. Mol. Plant Pathol..

[cit114] Berni R., Luyckx M., Xu X., Legay S., Sergeant K., Hausman J.-F., Lutts S., Cai G., Guerriero G. (2018). Environ. Exp. Bot..

[cit115] Khan M. N., Mobin M., Abbas Z. K., AlMutairi K. A., Siddiqui Z. H. (2017). Plant Physiol. Biochem..

[cit116] Zhao J., Hu Q., Guo Y.-Q., Zhu W.-H. (2001). Plant Sci..

[cit117] Phukan U. J., Jeena G. S., Shukla R. K. (2016). Frontiers in Plant Science.

[cit118] Kohan-Baghkheirati E., Geisler-Lee J. (2015). Nanomaterials.

[cit119] Kumari M., Khan S. S., Pakrashi S., Mukherjee A., Chandrasekaran N. (2011). J. Hazard. Mater..

[cit120] Ma C., Rui Y., Liu S., Li X., Xing B., Liu L. (2015). Sci. Rep..

[cit121] Sharma P., Jha A. B., Dubey R. S., Pessarakli M. (2012). J. Bot..

[cit122] Montillet J.-L., Chamnongpol S., Rustérucci C., Dat J., Van De Cotte B., Agnel J.-P., Battesti C., Inzé D., Van Breusegem F., Triantaphylides C. (2005). Plant Physiol..

[cit123] Møller I. M., Kristensen B. K. (2004). Photochem. Photobiol. Sci..

[cit124] Dimkpa C. O., McLean J. E., Latta D. E., Manangón E., Britt D. W., Johnson W. P., Boyanov M. I., Anderson A. J. (2012). J. Nanopart. Res..

[cit125] Zhang P., Ma Y., Zhang Z., He X., Li Y., Zhang J., Zheng L., Zhao Y. (2015). Nanotoxicology.

[cit126] Montes A., Bisson M. A., Gardella Jr J. A., Aga D. S. (2017). Sci. Total Environ..

[cit127] Dey P., Das N. (2013). Int. J. Pharm. Pharm. Sci..

[cit128] Basiuk E. V., Ochoa-Olmos O. E., De la Mora-Estrada L. F. (2011). J. Nanosci. Nanotechnol..

[cit129] Bao W., Wang J., Wang Q., Hare D., Wan Y. (2015). Sci. Rep..

[cit130] Verma S. K., Das A. K., Patel M. K., Shah A., Kumar V., Gantait S. (2018). Sci. Total Environ..

[cit131] Kim D. H., Gopal J., Sivanesan I. (2017). RSC Adv..

[cit132] Lee W. M., An Y. J., Yoon H., Kweon H. S. (2008). Environ. Toxicol. Chem..

